# Down syndrome and outcomes in critically ill pediatric patients

**DOI:** 10.3389/fped.2024.1483944

**Published:** 2025-01-24

**Authors:** José Tantaleán Da Fieno, Rosa León Paredes, Patricia Palomo Luck, Carlos Del Águila Villar, Emiliana Rizo Patrón

**Affiliations:** ^1^ Faculty of Human Medicine, Universidad Nacional Federico Villarreal, Lima, Peru; ^2^ Intensive Care Unit, Instituto Nacional de Salud del Niño, Lima, Peru; ^3^ Nutrition Services, Instituto Nacional de Salud del Niño; Department of Nutrition, Universidad Nacional Mayor de San Marcos, Lima, Peru; ^4^ Department of Physiology, Faculty of Human Medicine, Universidad Nacional Federico Villarreal; Endocrinology Services, Instituto Nacional de Salud del Niño, Lima, Peru; ^5^ Faculty of Human Medicine, Universidad Peruana Cayetano Heredia, Lima, Peru

**Keywords:** Down syndrome, pediatrics, critical care, cohort study, mechanical ventilation, infections

## Abstract

**Introduction:**

Information regarding children with Down syndrome (DS) in the Pediatric Intensive Care Unit (PICU) is limited and conflicting. We aimed to investigate the association between DS and clinical outcomes in pediatric patients admitted to the PICU at the Instituto Nacional de Salud del Niño (National Institute for Child Health, INSN for the acronym in Spanish) and to assess nutritional status within the study cohort.

**Methods:**

This study involved the secondary analysis of a database. We included patients consecutively admitted to INSN, a tertiary care children's hospital in Lima, Peru. We collected demographic data, clinical characteristics, and nutritional status using standardized tables. The outcomes assessed included mortality, length of stay, duration of mechanical ventilation (DMV), ventilator-free days (VFD), and healthcare-associated infections (HAI). We applied Cox regression and Poisson regression analyses to explore the relationship between Down syndrome and clinical outcomes, providing both crude and adjusted results.

**Results:**

A total of 525 children (average age 71.3 months, range 1–218 months) were analyzed. Children with DS were younger and had a higher prevalence of comorbidities, congenital heart disease, and underweight. Both crude bivariate and multivariate analyses demonstrated that children with DS had higher mortality rates, increased frequency of HAIs, longer DMV, longer PICU stay, and fewer VFD. Adjusted multivariate analysis revealed that children with DS had a significantly higher risk of developing HAIs (RR 2.95; 95% CI 1.10, 7.87) and longer DMV (RR 1.43; 95% CI 1.24, 1.65). Among the 525 children, underweight was associated with increased risk of mechanical ventilation (MV) use and longer DMV.

**Discussion:**

Critically ill children with DS are at increased risk of developing HAIs and longer DMV. In all 525 children, underweight is associated with higher risk of MV use and longer duration of MV.

## Introduction

Down syndrome (DS) is the most common chromosomal disorder, often accompanied by cardiac, respiratory, and gastrointestinal anomalies that increase the risk of hospitalization (1–4). Despite improvements in life expectancy over recent decades (2, 3), morbidity in children with DS has gained greater prominence, leading to increased hospitalizations and admissions to Pediatric Intensive Care Units (PICU) (2–5). Fifty percent of these children are hospitalized at least once before the age of three (4).

Studies focusing on critically ill children with DS are scarce. Two investigations using large PICU databases compared outcomes between children with and without DS (6, 7), and a retrospective study in a PICU analyzed the clinical course and mortality among children with DS (8). Other studies have examined children with DS in specific critical conditions such as cardiac surgery (9, 10), sepsis (11), acute lung injury (12), or Respiratory Syncytial Virus infection (RSV) (13, 14).

Respiratory problems are frequent in children with DS (3, 4, 7, 8). In children with pneumonia, a DS diagnosis is associated with higher risks of hospitalization (39.4% vs. 13.9%) and ICU admission (5). Children with DS and RSV infection have a greater risk of hospitalization (14), longer hospital stays, and worse outcomes compared to those without DS (13). DS is a risk factor for death in children with severe RSV infection (15). Hospitalized children with DS often exhibit a more severe clinical course (11, 12), requiring increased PICU utilization, organ support, longer hospital stays, and facing higher mortality risks (2, 3). The propensity for autoimmunity in DS, characterized by persistent cytokine elevation, hyperactivation of CD4T cells, and continuous activation of B cells, may disrupt immune tolerance and predispose these children to severe infections (16).

The clinical course of critically ill children with DS is variable. In a cohort of 10,477 children with DS, a higher likelihood of PICU admission was observed, with similar mortality rates compared to children without DS (6). Among 1,278 children with DS in PICUs, there was a greater need for life support than expected based on initial severity, yet *lower* crude mortality compared to children without DS (7). Both studies (6, 7) highlighted the prevalence of cardiovascular problems, particularly in children with DS. Two studies examined the outcomes of critically ill children with DS who did not undergo cardiac surgery. In 66 children with DS in PICUs, mortality was nearly three times higher than in children without DS (39.4% vs. 14%); sepsis and nosocomial infections were identified as risk factors for death (8). Another study on septic children with DS reported higher mortality, more frequent mechanical ventilation (MV) use, more frequent PICU admissions, and a higher incidence of infections compared to other children (11). The outcomes of children with DS undergoing cardiac surgery also vary: depending on the type of surgery, hospital stays and mortality rates can either be greater or lower than those of children without DS (9, 10).

Similarly, information on nutritional status in critically ill children with DS is limited and inconsistent. One study found no differences in nutritional status between children with and without DS; among those with DS, 6% were obese and none were malnourished (13). In children undergoing cardiac surgery, the weight z-score in children with DS was notably lower than in those without the syndrome (−2.1 vs. −0.9) (10). It remains unknown whether nutritional status in critically ill children with DS affects their outcomes.

While data suggest a consistent higher risk of hospitalization among children with DS, outcomes in the PICU exhibit mixed results, both in patients with medical conditions and in cardiac surgery patients. Due to frequent anomalies across various organs and immune system disturbances (16, 17), critically ill children with DS may experience a more severe or unfavorable clinical course (18) compared to critically ill children without DS. In this study, we analyze children with DS compared to those without the syndrome, examining their association with mortality, length of PICU stays, development of healthcare-associated infections (HAIs), and use of invasive MV in a mixed PICU. Additionally, we assessed nutritional status and its association with these outcomes across the study population.

## Materials and methods

This publication adheres to the Strengthening the Reporting of Observational Studies in Epidemiology (STROBE) guidelines.

### Design, setting, and participants

We conducted a secondary analysis of a prospective cohort study database spanning from April 2018 to April 2020 at PICU of the Instituto Nacional de Salud del Niño (National Institute for Child Health, INSN for the acronym in Spanish). This previous study included patients consecutively admitted to the PICU and aimed to assess the association between nutritional status and four clinical outcomes: mortality, length of stay, occurrence of HAIs, and duration of MV (DMV). This study excluded patients with Down syndrome (DS), and the manuscript is currently in preparation.

The INSN PICU admits all types of medical and surgical patients under 18 years old, excluding neonates and those from post-cardiac surgery. Data were directly obtained from patients or their medical records. We included all consecutively admitted patients during the study period, with and without DS. Demographic data were recorded for all admissions, such as age, sex, severity of illness, admission diagnosis, and presence of comorbidities. Exclusion criteria encompassed children under 2 years with a history of prematurity, skeletal malformations, edema upon admission, brain death, or death within ≤24 h of PICU admission, as well as extreme nutritional data (*z* score <−6). We excluded early mortality as it is a potential confounder that could bias the incidence of mortality, as it may be influenced by factors unrelated to the presence of Down syndrome, such as extreme severity. Only the first admission was included.

### Study variables

We recorded admission diagnoses and comorbidities and evaluated patients daily during their PICU stay. Hospital discharge or death was tracked through the INSN's electronic system. Anthropometric measurements were taken within the first 48 h of admission, with no routine subsequent nutritional assessments. Severity was assessed using the PRISM (Pediatric Risk of Mortality Score) scale (19).

Nutritional status evaluation utilized three tools: PediTools (20) and WHO Anthro (21) for children <1 year; WHO Anthro and WHO AnthroPlus (22) for children ≥1 year, tailored for those under and over 5 years, respectively. Criteria and classification of nutritional status for children with and without DS are shown in Table 1. Children with DS were assessed using criteria outlined in a Technical Standard from the Chilean Ministry of Health (26).

**Table 1:** Criteria for nutritional status classification.

	Age group	Indicator	Cut-off values	References
DS	<1 year	W/A W/L	UW: <10th percentile OW: ≥+1 SD N: any other value	Zemel W/A chart (23) WHO 2006 growth curves (24)
	≥1 year to <5 years	W/L	UW: ≤−2SD N: >−2SD to <+1SD OW: ≥+1 SD	WHO 2006 growth curves (24)
	≥5 years	BMI/A	UW: ≤−2SD N: >−2SD to <+1SD OW: ≥+1 SD	WHO 2007 growth curves (25)
No DS	<1 year ≥1 year to <2 years ≥2 years	W/A W/L BMI/A	For all indicators: UW: <−2SD N: ≥−2SD to <+1SD OW: ≥+1 SD	WHO 2006 growth curves (24) WHO 2007 growth curves (25)

DS, Down Syndrome; W/A, weight for age; W/L, weight for length; BMI/A, body mass index for age; UW, underweight; N, normal weight; OW, overweight.

Postoperative (PO) diagnosis was defined for scheduled surgeries, and medical diagnosis for all other cases. Medical diagnoses were classified as respiratory, sepsis, neurological, cardiovascular, and others. The presence of comorbidity was recorded in two categories: heart disease and other comorbidities.

### Outcomes

Mortality was evaluated up to 60 days, while PICU and hospital stays were measured, focusing solely on PICU stay. For bivariate analysis, all patient stays were included, whereas for multivariate analyses of duration of MV (DMV) and PICU stay, only survivors were included. HAIs and DMV were investigated daily during PICU stay. We evaluated the use of invasive MV using DMV and ventilator-free days (VFD). If a child was intubated upon PICU admission, DMV was considered from PICU admission until extubation; if reintubated <48 h post-extubation, both periods were counted as a single DMV episode. For intubated patients, VFD were calculated as 28 days minus days on invasive MV; if a child died before 28 days or remained on MV beyond 28 days, it was recorded as 0 (zero). Non-invasive ventilation (NIV) use was noted, but all MV-related analyses (MV use, DMV, and VFD) were conducted only in children on invasive MV. Active surveillance was conducted to detect three HAIs (bloodstream infection, ventilator-associated pneumonia, or urinary tract infection), based on criteria by Horan et al. (27); these diagnoses were confirmed by the Epidemiological Surveillance Unit of INSN.

### Statistical analysis

Efforts were made to minimize the impact of bias. All patients were from the same population and objective data was collected prospectively. Extreme severity admissions were limited by excluding patients who died within the first 24 h; VFDs mitigated the impact of early deaths. Other potential confounders such as PRISM score, comorbidity, diagnosis, and PO status were controlled in adjusted analysis.

Normal distribution of the dataset was verified using the Shapiro-Wilk test, and appropriate descriptive analyses were proposed based on these results. To assess the association of DS with outcomes (mortality, HAIs, MV, and stay), relative risks were calculated using a Poisson regression with a generalized linear model (GLM), Poisson family, and logarithmic link function, employing robust standard errors. DMV and VFD outcomes were analyzed using zero-inflated Poisson (ZIP) regression models. For multivariate analyses, we selected the independent variables showing ≤0.1 statistical significance in bivariate analyses, in addition to age and sex. Ninety-five percent confidence intervals are reported for each association measure. Due to the small number of children with DS, we did not assess the interaction of DS with nutritional status. STATA version 15.0 (StataCorp. 2017. Stata Statistical Software: Release 15. College Station, TX, USA) was used for statistical analyses.

We used the Kaplan-Meier survival analysis for assessing mortality, VFD, and stay outcomes. Kaplan-Meier curves were generated to estimate and visualize survival functions for patients with and without DS. Time-to-event data were censored for individuals who did not experience the event by the study end or were lost to follow-up. Log-rank tests were applied to compare survival distributions between patients with and without DS.

### Ethical considerations

The Institutional Ethics Committee of INSN approved this study under code PI-44/22. It is an observational study utilizing a database. Study procedures preserved patient confidentiality, integrity, and fundamental rights.

## Results

**Figure 1:**
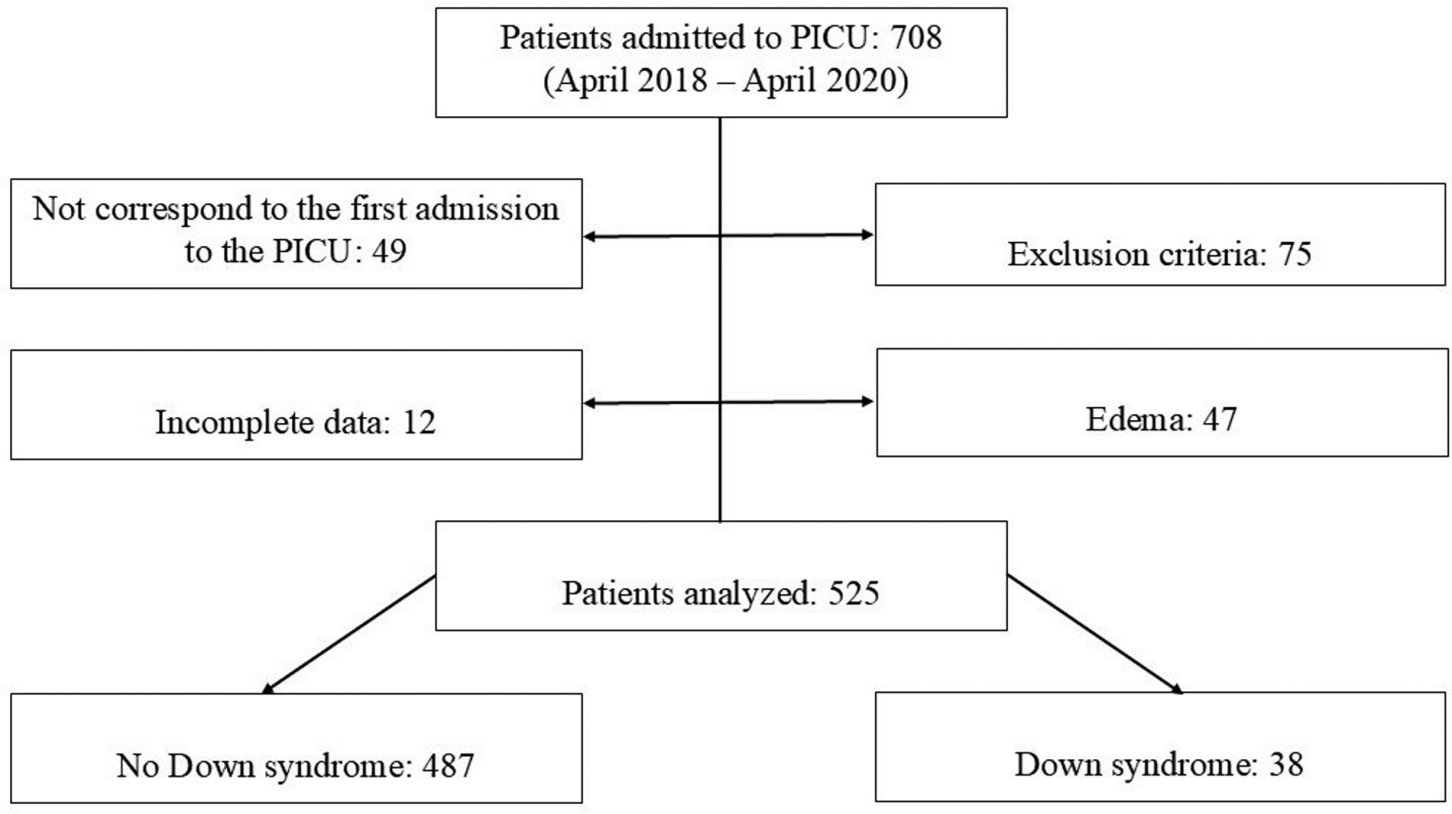
Study flowchart. PICU: Pediatric Intensive Care Unit.

There were 708 PICU admissions, with 54 (7.6%) corresponding to children with DS. After excluding readmissions, duplicate records, incomplete data, and applying exclusion criteria, 525 children, 38 with DS and 487 without DS, comprised the analyzed population (Figure 1). Demographic and clinical characteristics of both groups are shown in Table 2.

**Table 2:** Characteristics of children with and without Down syndrome.

Characteristic	All (525)	DS (*n* = 38)	No DS (*n* = 487)	*p*
Age in months, median (IQR)	55 (9–128)	8 (3–16)	64 (11–132)	**<0** **.** **001** ^a^
Male sex, *n* (%)	307 (58.5)	18 (47.4)	289 (59.3)	0.149
PRISM score, median (IQR)^b^	7 (4–12)	7 (4–13)	7 (4–11)	0.841^a^
Diagnosis, *n* (%)				0.002
PO	222 (42.3)	7 (18.4)	215 (44.1)	
Medical	303 (57.7)	31 (81.6)	272 (55.9)	
Respiratory diagnosis, *n* (%)	119 (22.7)	17 (44.7)	102 (21.0)	**0** **.** **001**
Sepsis, *n* (%)	52 (9.9)	6 (15.8)	46 (9.5)	0.252^c^
Any comorbidity, *n* (%)^d^	143 (27.2)	32 (84.2)	111 (22.8)	**<0** **.** **001**
Heart Disease, *n* (%)^e^	46 (8.8)	24 (63.2)	22 (4.5)	**<0** **.** **001** ^c^
Other comorbidities	107 (20.4)	8 (21.1)	99 (20.1)	0.915
Nutritional status, *n* (%)				
Underweight	87 (16.6)	23 (60.5)	64 (13.1)	
Normal weight	325 (61.9)	14 (36.8)	311 (63.9)	
Overweight	113 (21.5)	1 (2.6)	112 (23.0)	**<0** **.** **001**

DS, Down syndrome; PO, postoperative; IQR, interquartile range.

Significant values are shown in bold.

^a^ Mann–Whitney *U* test.

^b^ Results based on available valid data (total = 438, DS = 35, no DS = 403).

^c^ Fisher's exact test.

^d^ Includes congenital heart diseases.

^e^ Includes congenital heart disease as admission diagnosis and as a comorbidity; in children without DS, includes congenital heart diseases and other cardiovascular problems.

Children with DS were younger and had a higher incidence of heart disease, the most frequent comorbidity. The frequency of other comorbidities was similar in both groups. Among the 24 children with DS and heart disease (congenital heart disease in all), 14 (58%) developed heart failure. Nearly half of the children with DS (45%) were admitted for respiratory issues, contrasting with 21% of other children. The frequency of PO cases was lower in children with DS (18.4% vs. 44.1% in children without DS), all comprising abdominal surgery (congenital diaphragmatic hernia in 5 cases and intestinal obstruction in 2 cases). There were 23 (60.5%) underweight children with DS, of whom 7 died, 14 children with normal weight (5 deceased), and one case of obesity (0 deaths). The prevalence of underweight was higher in children with DS compared to the rest of the population (60.5% vs. 13.1%).

### Bivariate analysis

Table 3 displays the bivariate analysis of outcomes for children with and without DS. In children with DS, mortality, PICU stay days, DMV, and HAI frequency were significantly higher, while VFD were significantly lower compared to children without DS. Children with DS had nearly a fourfold higher risk of death; children with DS with congenital heart disease had a mortality rate of 45.8% (11/24), whereas in children with DS without heart disease, mortality was 21.4% (3/14) (data not shown). The risk of acquiring HAIs was nearly five times higher in children with DS, with the majority (11/12) being ventilator-associated pneumonia. Longer DMV and fewer VFD in children with DS indicate longer duration of invasive respiratory support relative to children without DS. NIV was used with 44.7% of children with DS and 15.6% of children without DS (data not shown).

**Table 3:** Bivariate analysis of outcomes in children with and without Down syndrome.

	All (525)	DS (*n* = 38)	No DS (*n* = 487)	*p*
Mortality, *n* (%)	53 (10.1)	12 (31.6)	41 (8.4)	**<0** **.** **001** ^a^
PICU Length of Stay, median (IQR)	6 (3–14)	13 (7–20)	6 (3–13)	**<0** **.** **001** ^b^
Use of IMV, *n* (%) ^c^^“a”^	410 (78.8)	34 (89.5)	376 (78.8)	0.096
DMV in days, median (IQR) ^c^^“b”^	2 (0–7)	5 (2–17)	2 (0–7)	**0** **.** **001** ^b^
VFD, median (IQR)^c^^“c”^	24 (13–27)	19 (9–25)	25 (14–27)	**0** **.** **020** ^b^
HAI, *n* (%)^c^^“d”^	44 (8.4)	12 (31.6)	32 (6.6)	**<0** **.** **001** ^a^

HAI, Healthcare-Associated Infection; IMV, Invasive Mechanical Ventilation; DMV, Duration of Mechanical Ventilation; VFD, Ventilator-Free Days.

Significant values are shown in bold.

^a^ Fisher's exact test.

^b^ Mann–Whitney *U* test.

^c^ Results are based on available valid data (^a^520; ^b^519; ^c^410; ^d^522). The analysis of DMV includes patients with no use of MV, for whom 0 days were assigned.

**Figure 2:**
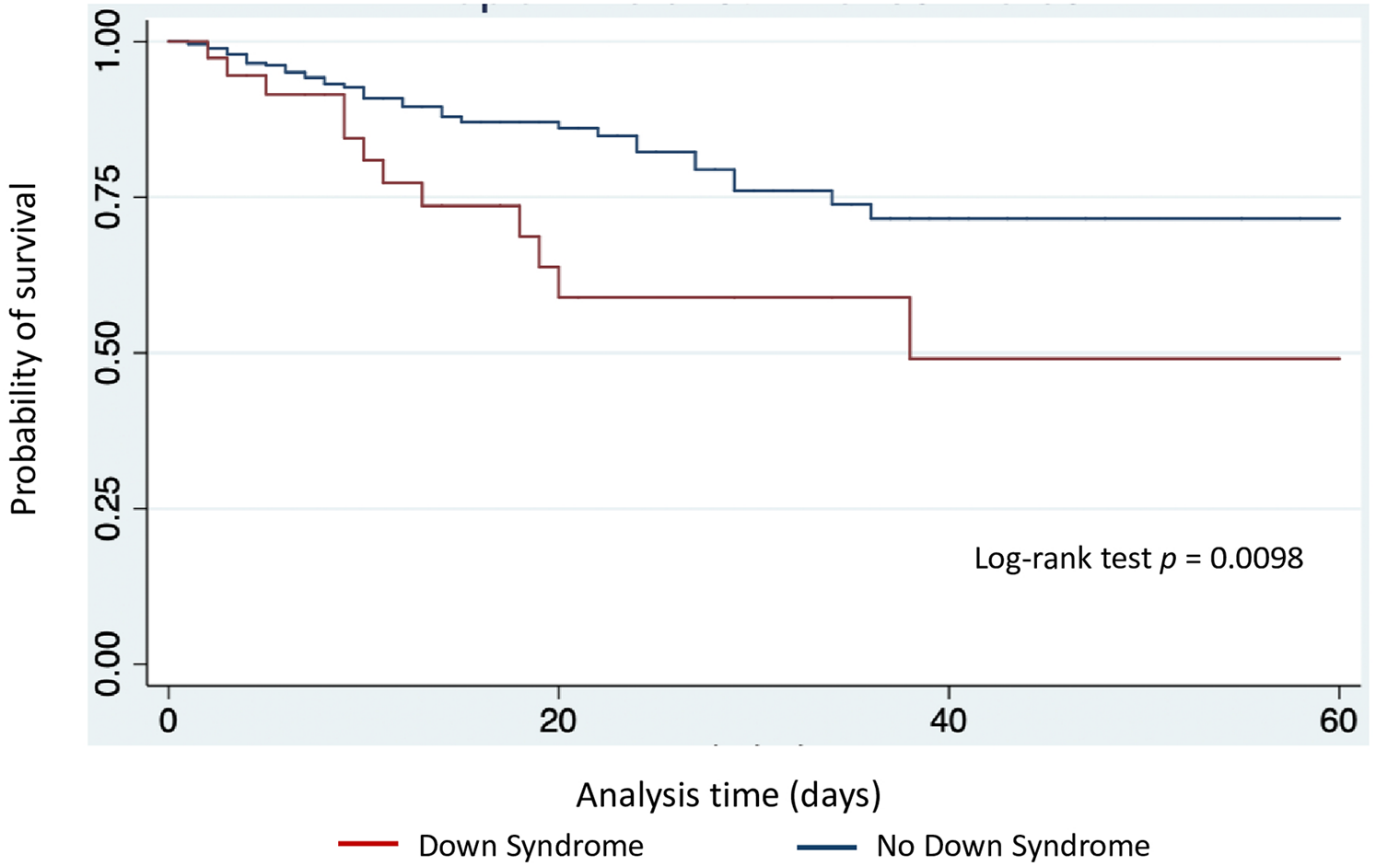
Kaplan-Meier estimates of survival at 60 days, according to presence of Down syndrome.

**Figure 3:**
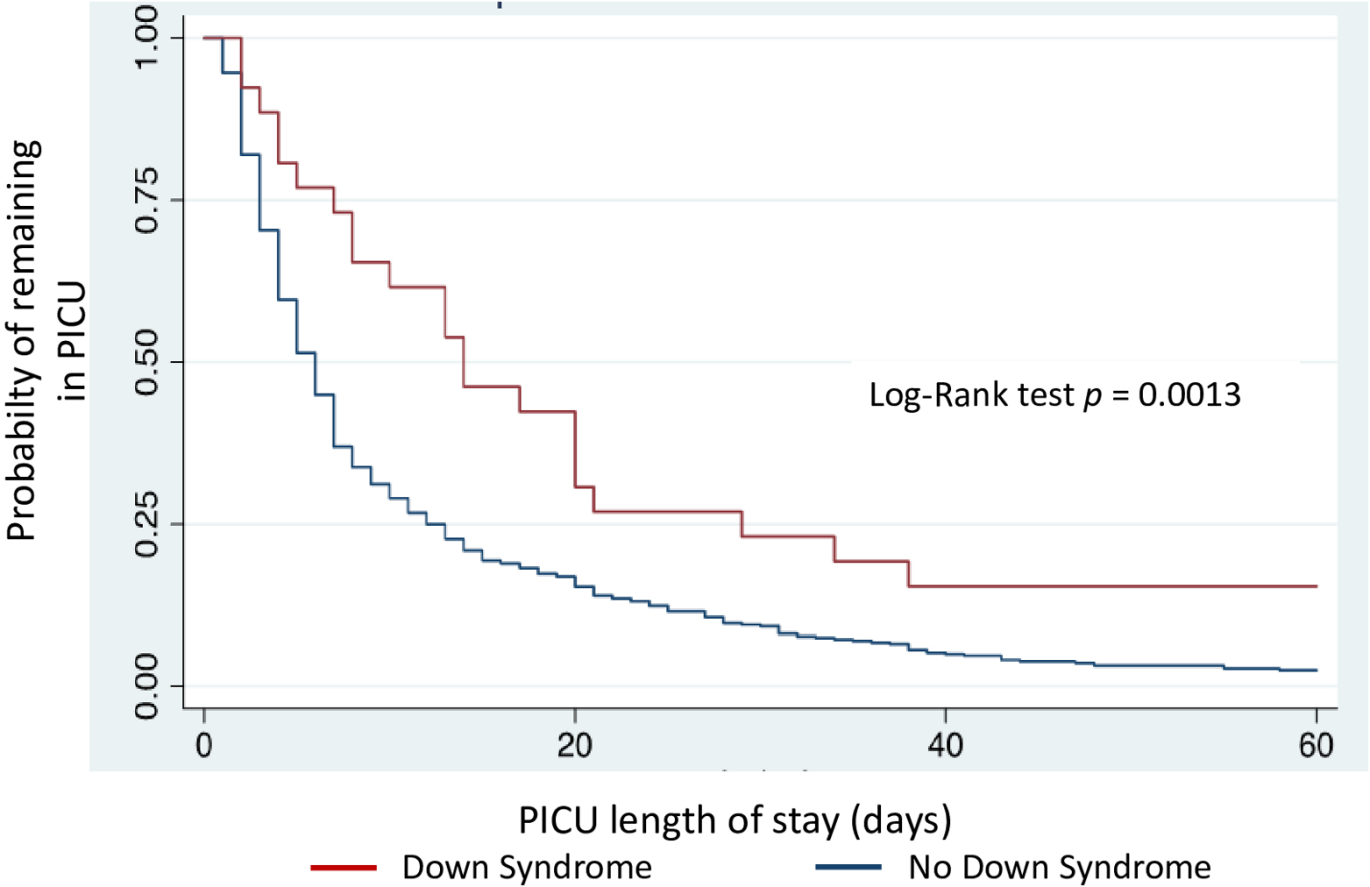
Cumulative percentage of patients remaining in PICU, according to presence of Down syndrome. PICU: Pediatric Intensive Care Unit.

**Figure 4:**
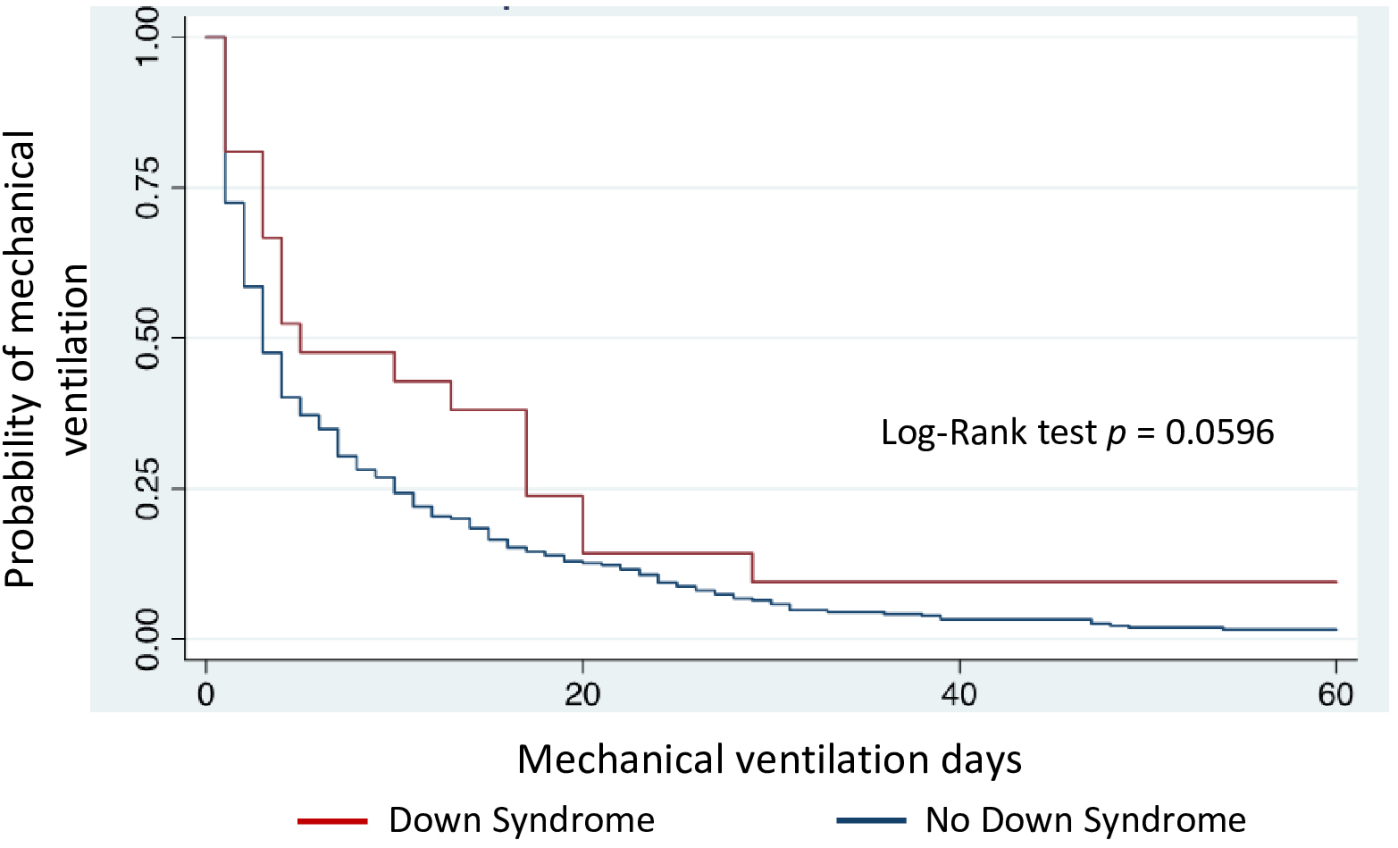
Duration of mechanical ventilation in children with and without Down syndrome.

Kaplan-Meier curves demonstrate higher mortality risk and longer PICU stay in children with DS (log-rank test, *p* = 0.0098 and *p* = 0.0013, respectively) (Figures 2, 3). DMV values did not differ between children with and without DS (*p* = 0.0596) (Figure 4), nor in VFD (*p* = 0.109).

### Multivariate analysis

Multivariate analyses were adjusted for age, sex, PRISM score, nutritional status, heart disease, and PO and respiratory diagnoses. Unadjusted analyses indicated higher mortality, increased HAI frequency, longer DMV, longer PICU stay, and fewer VFD in children with DS. However, in adjusted analyses, only HAI development and DMV were significantly associated with DS, whereas PICU stay showed marginal association (Tables 4–9). For each outcome, alongside their association with DS, we show associations with other variables significant in bivariate analysis.

**Table 4:** Multivariate analysis of mortality at 60 days.

Characteristics	Unadjusted RR (CI 95%)	*p*-value	Adjusted^a^ RR (CI 95%)	*p*-value
Down Syndrome	**3.75 (2.16, 6.52)**	**<0.001**	1.49 (0.56, 3.94)	0.416
PRISM Score			**1.10** **(1.06, 1.15)**	**<0** **.** **001**
Nutritional status				
Underweight			Reference	0.662
Normal weight			0.85 (0.42, 1.73)	0.662
Overweight			0.51 (0.19, 1.41)	0.194
Postoperative status			0.80 (0.38, 1.66)	0.507
Respiratory diagnosis			1.29 (0.69, 2.42)	0.427
Heart disease			1.40 (0.60, 3.29)	0.436

All *p*-values were obtained from a Poisson regression model with robust standard errors.

Significant values are shown in bold.

^a^ Poisson regression. Adjusted for age, sex, PRISM score, nutritional status, cardiac condition, Postoperative and respiratory diagnoses.

**Table 5:** Multivariate analysis of healthcare-associated infection.

Characteristics	Unadjusted RR (CI 95%)	*p*-value	Adjusted^a^ RR (CI 95%)	*p*-value
Down Syndrome	**4.78** **(2.68, 8.50)**	**<0.001**	**2.95** **(1.10, 7.87)**	**0** **.** **031**
PRISM Score			**1.05** **(1.01, 1.10)**	**0** **.** **016**
Nutritional status				
Underweight			Reference	0.626
Normal weight			0.83 (0.40, 1.74)	0.626
Overweight			0.59 (0.21, 1.63)	0.308
Postoperative status			**0.13** **(0.03, 0.52)**	**0** **.** **004**
Respiratory diagnosis			1.88 (0.98, 3.58)	0.056
Heart disease			0.96 (0.41, 2.25)	0.934

All *p*-values were obtained from a Poisson regression model with robust standard errors.

Significant values are shown in bold.

^a^ Poisson regression. Adjusted for age, sex, PRISM score, nutritional status, cardiac condition, Postoperative and respiratory diagnoses.

**Table 6:** Multivariate analysis of mechanical ventilation use.

Characteristics	Unadjusted RR (CI 95%)	*p*-value	Adjusted^a^ RR (CI 95%)	*p*-value
Down Syndrome	**1.15** **(1.02, 1.29)**	**0.024**	1.04 (0.85, 1.26)	0.731
PRISM Score			**1.03** **(1.02, 1.04)**	**<0** **.** **001**
Nutritional status				
Underweight			Reference	
Normal weight			**0.86** **(0.79, 0.95)**	**0.003**
Overweight			**0.80** **(0.70, 0.92)**	**0.002**
Postoperative status			**1.48** **(1.31, 1.67)**	**<0** **.** **001**
Respiratory diagnosis			1.14 (0.98, 1.32)	0.088
Heart disease			1.09 (0.91, 1.31)	0.336

All *p*-values were obtained from a Poisson regression model with robust standard errors.

Significant values are shown in bold.

^a^ Poisson regression. Adjusted for age, sex, PRISM score, nutritional status, cardiac condition, Postoperative and respiratory diagnoses.

**Table 7:** Multivariate analysis of duration of mechanical ventilation.^a^

Characteristics	Unadjusted RR (CI 95%)	*p*-value	Adjusted^b^ RR (CI 95%)	*p*-value
Down Syndrome	**1.77** **(1.58, 1.98)**	**<0.001**	**1.43** **(1.24, 1.65)**	**<0** **.** **001**
PRISM Score			**1.01** **(1.01, 1.02)**	**<0** **.** **001**
Nutritional status				
Underweight			Reference	
Normal weight			**0.86** **(0.78, 0.95)**	**0.002**
Overweight			**0.67** **(0.59, 0.78)**	**<0.001**
Postoperative status			**0.35** **(0.31, 0.39)**	**<0** **.** **001**
Respiratory diagnosis			**1.41** **(1.29, 1.54)**	**<0** **.** **001**
Heart disease			**0.78** **(0.68, 0.90)**	**<0** **.** **001**

*p*-values were derived from a zero-inflated Poisson (ZIP) regression model.

Significant values are shown in bold.

^a^ Only in survivors.

^b^ Zero inflated Poisson regression. Adjusted for age, sex, PRISM score, nutritional status, cardiac condition, Postoperative and respiratory diagnoses.

**Table 8:** Multivariate analysis of ventilator-free days.

Characteristics	Unadjusted RR (CI 95%)	*p*-value	Adjusted^a^ RR (CI 95%)	*p*-value
Down Syndrome	**0.86** **(0.79, 0.93)**	**<0.001**	0.96 (0.86, 1.08)	0.537
PRISM Score			0.99 (0.99, 1.0)	<0.001
Nutritional status				
Underweight			Reference	
Normal weight			1.03 (0.96, 1.10)	0.441
Overweight			**1.09** **(1.00, 1.18)**	**0.044**
Postoperative status			**1.15** **(1.08, 1.23)**	**<0** **.** **001**
Respiratory diagnosis			**0.86** **(0.80, 0.94)**	**<0** **.** **001**
Heart disease			1.05 (0.95, 1.18)	0.342

*p*-values were derived from a zero-inflated Poisson (ZIP) regression model.

Significant values are shown in bold.

^a^ Zero inflated Poisson regression. Adjusted for age, sex, PRISM score, nutritional status, cardiac condition, Postoperative and respiratory diagnoses.

**Table 9:** Multivariate analysis of ICU length of stay.^a^

Characteristics	Unadjusted RR (CI 95%)	*p*-value	Adjusted^b^ RR (CI 95%)	*p*-value
Down Syndrome	**2.11** **(1.36, 3.28)**	**0.001**	1.51 (0.99, 2.31)	0.057
PRISM Score			**1.03** **(1.02, 1.05)**	**<0** **.** **001**
Nutritional status				
Underweight			Reference	
Normal weight			0.78 (0.56, 1.09)	0.154
Overweight			**0.62** **(0.41, 0.92)**	**0.016**
Postoperative status			**0.51** **(0.39, 0.68)**	**<0** **.** **001**
Respiratory diagnosis			**1.51** **(1.10, 2.05)**	**0** **.** **010**
Heart disease			0.87 (0.57, 1.33)	0.522

*p*-values were derived from a zero-inflated Poisson (ZIP) regression model.

Significant values are shown in bold.

^a^ Only in survivors.

^b^ Zero inflated Poisson regression. Adjusted for age, sex, PRISM score, nutritional status, cardiac condition, Postoperative and respiratory diagnoses.

Mortality was higher in patients with DS in the unadjusted analysis, but adjusted analysis showed no statistical significance (Table 4). PRISM score significantly increased mortality risk, whereas nutritional status did not influence death risk. Each 1-point increase in PRISM was associated with a 10% higher mortality risk (RR: 1.10; CI 1.06, 1.15). Isolated heart disease as a comorbidity did not associate with higher mortality. We examined whether the association between Down syndrome and mortality varied based on the timing of the event (early vs. late deaths) and found no significant differences (data not shown).

Children with DS had a threefold higher risk of developing HAIs (RR 2.95; CI 1.10–7.87) (Table 5). PO status was associated with lower HAI frequency (RR 0.13; CI 0.03–0.52), whereas higher PRISM score was associated with increased frequency of HAI. Nutritional status, respiratory diagnosis, and comorbidity were not associated with the occurrence of HAI.

No increased risk of MV use was found in children with DS (RR 1.04; CI 0.85–1.26) (Table 6). Children with normal weight or overweight had lower risk of MV use, with higher incidence of use of invasive respiratory support in underweight children. PO diagnosis and higher PRISM score strongly correlated with MV use, whereas comorbidity, respiratory diagnosis, and heart disease were not associated.

Children with DS had longer DMV (RR 1.43; CI 1.24, 1.65), indicating that they were more likely to remain on mechanical ventilation compared to children without DS; similarly, among 525 children, underweight and respiratory diagnosis significantly correlated with longer DMV, whereas PO patients and children with cardiovascular issues had shorter DMV (Table 7).

The presence of DS was associated with fewer VFD in the unadjusted analysis, but significance was lost in the adjusted analysis, while respiratory diagnosis and higher PRISM score significantly reduced VFD. PO patients had more VFD, indicating shorter DMV. No association was found between VFD and nutritional status, but overweight children had more VFD compared to underweight children. Heart disease did not correlate with VFD (Table 8).

The presence of DS was not related to longer **PICU stay** in the adjusted analysis, although it had marginally statistical significance (RR 1.51; CI 0.99, 2.31; *p* = 0.057). Respiratory diagnosis was associated with a longer stay, whereas PO patients had a shorter stay. Heart disease did not correlate with PICU stay among the study population (Table 9).

## Discussion

In this study, we observed that critically ill children with DS have a higher risk of developing HAIs and longer DMV compared to children without the syndrome. While univariate and crude multivariate analyses initially associated DS with all outcomes, adjusted analysis revealed that only HAIs (RR 2.95; CI 1.10, 7.87) and MV duration (RR 1.43; CI 1.24, 1.65) remained significantly associated with DS. Regarding nutritional aspects, 60% of children with DS were underweight, and among the 525 children included, underweight status was associated with increased risk of MV use and longer DMV.

Immunological alterations in children with DS may predispose them to higher frequencies of **HAIs** and more severe infections (16–18). Respiratory tract infections are a common cause of hospitalization in children with DS (4, 28), attributed to dysfunction in T and B lymphocytes, neutrophils, monocytes, and altered complement function (17). Although the pathogenesis of HAIs differs from that of community-acquired infections, it is noteworthy that in our study, 11 out of 12 HAIs were ventilator-associated pneumonias. Surprisingly, most publications on critically ill children with DS have not evaluated HAIs, and studies that have done so report conflicting findings. For instance, in children with DS undergoing surgery for atrial septal defects and ventricular septal defects, a higher frequency of infections was observed compared to children without DS. However, in children undergoing Tetralogy of Fallot repair, infection frequencies were similar in both groups (10). Our findings of a threefold higher risk of HAIs suggest that critically ill children with DS should be closely monitored for the development of serious infections.

It is known that hospital stays for children with DS can be up to three times longer than those for children without DS (29). However, studies on **PICU stays** of critically ill children show conflicting results. In critically ill children with DS, longer stays (7) as well as shorter stays (6) have been described. Studies in children undergoing cardiac surgery show that DS may influence length of stay depending on the type of surgery; stays are longer compared to those of children without DS in cases of atrial and ventricular septal defects (9, 10) and Tetralogy of Fallot repair (10), but shorter in mitral valve surgery (9). The differences observed have been attributed to preoperative risk factors and PO complications (10). Because our PICU does not admit children who have undergone cardiac surgery, the longer stay of children with DS observed in our unadjusted analysis is consistent with findings from studies conducted in a similar population (8). The length of stay for children with DS in the aforementioned study was 13.3 days, which is nearly identical to what we observed in our results. However, in adjusted analysis, the longer stay in DS showed only marginal statistical significance (RR 1.51; CI 0.99, 2.31).

PICU stay may also be influenced by DMV, as will be explained later. In this study, patients with DS had longer DMV and a trend towards longer PICU stays, although this did not reach statistical significance. The use of NIV may also prolong PICU stay as its use is preferred in areas with higher levels of specialized personnel and equipment. NIV use in children with DS was nearly three times higher, but we did not analyze this data.

**Mortality** in critically ill children with DS has shown inconsistent findings, but appears consistently higher when studies do not predominantly include children who have undergone cardiac surgery. In a general PICU, mortality in children with DS was three times higher than in children without DS admitted during the same time period (8). In septic children with DS, adjusted mortality was 30% higher than in the rest of the population (11), and in children with severe Respiratory Syncytial Virus infection, DS was a risk factor for mortality (15). Conversely, studies in PICUs with a population of children predominantly affected by cardiac and cardiovascular issues report similar (6) or lower (7) mortality in children with DS compared to those without DS. Likewise, in studies limited to children undergoing cardiac surgery, mortality rates in DS were higher, similar, or even lower depending on the type of surgery performed (9, 10).

In this study, the higher mortality among children with DS did not reach statistical significance in the adjusted model (RR 1.49; CI 0.56, 3.94), likely due to the small number of cases or covariable adjustments. Younger age, higher frequency of comorbidity or heart disease (30) and being underweight (31) have correlated with higher mortality. These conditions were present in this study, which could explain higher mortality among children with DS in crude analysis. As we previously described, studies that excluded cardiac surgery cases (8, 11, 15), akin to ours, have also associated DS with increased mortality. Certain conditions present in DS, such as anomalies in various organs and systems, particularly congenital heart disease (28), or immunological alterations in the innate and adaptive systems that facilitate a more severe evolution (17), may increase mortality.

We found higher mortality in children with DS with congenital heart disease compared to children without heart disease (45.8% vs. 21.4%) in descriptive and bivariate analyses, but adjusted regression analysis showed similar mortality in both groups. The lack of association between mortality and heart disease may be due to the small number of children with heart disease in both study groups; furthermore, among children without DS, cardiovascular diagnoses encompassed a diversity of conditions, with only a quarter having congenital heart disease, and nearly half involving hemorrhagic shock. Critical patients without heart disease may have a less unfavorable outcome than children with heart disease. The influence of immunological alterations in DS on mortality in our study remains speculative, as we did not investigate them.

Several studies have reported that the similar or lower mortality observed in critically ill children with DS transitions to higher mortality as the length of hospital stay increases (6, 7). The underlying reason for this phenomenon remains unclear, but it has been attributed to a defective immune response, to clinical decisions biased by the presence of DS, or to an adaptive response wherein mitochondria enter a state described as “hibernation” (7). In the latter scenario, at the onset of critical illness with organ failure due to oxygen delivery deficiency, mitochondrial biogenesis is activated to meet adenosine triphosphate demands; upon resolution of the causal illness, mitochondrial biogenesis facilitates organic function recovery (7, 32). Thus, the activation of mitochondrial biogenesis prevents premature death, while long-term survival depends on successful mitochondrial recovery (7). It is noteworthy that mitochondria in children with DS are predisposed to oxidative stress (2).

In contrast to our findings, which showed that the association between Down syndrome and mortality was consistent regardless of the timing of the event (early vs. late deaths), the study by Tibby et al. (7) reported that Down syndrome was associated with an increased risk of death exclusively after ten days of PICU stay. It should be noted that Tibby's study (7) compared more than 1,000 children with DS and more than 32,000 without DS. Due to the multiplicity of factors that can trigger death in critically ill children with DS, as well as the small number of cases, we were unable to perform an analysis with sufficient power to assess the association between a specific length of stay and mortality.

Adjusted analysis showed higher **DMV** in children with DS (RR 1.43; CI: 1.24, 1.65) (Table 7), but we found no association of DS with VFD (Table 8). These results indicate that the expected DMV for children with DS is 1.43 times greater than that for children without DS. Several characteristics in this population of children with DS may increase DMV and decrease VFD. Malnutrition in children increases DMV (33), and 60% of children with DS in this study were underweight. The association with VFD may have been lost in the adjusted analysis due to the inclusion of nutritional status. The apparent contradiction between the higher DMV and the similar amount of VFD observed in children with DS in the adjusted analysis may be due to the way these variables are calculated.

Additionally, hypotonia in children with DS (2, 29) and muscle weakness frequently observed in critical patients can affect respiratory system function, prolong the DMV, and increase PICU stay (34). The diaphragm is an important respiratory muscle, and malnourished children have a thinner diaphragm, negatively affecting patient outcomes, particularly those with lower respiratory infections (35). In adults, a thinner diaphragm is associated with longer DMV and higher mortality (36). Among the 525 children (including those with DS), underweight status was associated with increased MV use and longer DMV. These data indicate a greater need to prolong invasive respiratory support in children with DS who are also underweight.

Admissions corresponded to 7.6% of children with DS, which is higher than the 4.2% and 3.8% reported in studies of large databases (6, 7). The high frequency of underweight status among children with DS may have influenced the higher admission frequency, as other characteristics, such as age ≤2 years and high frequency of respiratory problems and congenital heart diseases, were similar to those described in other studies (6–8). 63.1% of children with DS in the study had heart disease, a figure comparable to the 54%–79% found in other critical children studies (6–8). Other probable reasons for higher admission rate include institutional policies and institutional policies and the scarcity of intermediate levels of care in other hospital areas, because when we conducted the study, the hospital did not have an intermediate care unit.

It is known that children with DS have a higher risk of PICU admission than children without the syndrome (4, 6, 7). Respiratory problems, especially pneumonia, are the most common reasons for ICU admission (4, 5, 13, 14, 28) This was also observed in our results, as nearly half of the children with DS were admitted due to these issues. Increased oxidative stress, greater susceptibility to infections, hypotonia, and immunological alterations (2, 17) appear to be the causes of the higher admission frequency. Similarly, frequent respiratory anomalies in DS (29, 37) could promote the higher incidence of Pediatric Acute Respiratory Distress Syndrome (PARDS) observed in children with DS on MV (12). It is notable that four out of 38 children with DS were admitted with PARDS, contrasting with the 3.2% incidence found in an international multicenter study in PICUs (38). It has been described that even cardiac, gastrointestinal, and immunological anomalies, which are also frequent in DS, may affect proper respiratory system function (29).

Most studies in critically ill children with DS have not assessed the nutritional aspect (6–9, 11, 12). The sole study presenting nutritional data focused on children with DS undergoing cardiac surgery, revealing a median weight z score lower than that of children without DS (−2.1 vs. −0.9) (10); however, it did not analyze whether this condition influenced outcomes such as mortality, length of stay, or infections. Nutritional status can impact the evolution of critically ill patients, albeit other factors typically coexist. As mentioned earlier, malnutrition may increase DMV (33), which is potentially exacerbated by muscle mass loss seen in critically ill patients (34), as it compromises respiratory muscle and diaphragm function (34, 35). With increased DMV, ICU stay tends to be longer, as observed in the present study. Various conditions, including the high frequency of malnutrition, DS-associated hypotonia, and muscle mass loss in critically ill children, may have synergistically contributed to longer DMV. The longer duration of MV in underweight children observed in the present study (Table 7) is consistent with the results of other studies (39, 40).

Conversely, a study on children with DS and Respiratory Syncytial Virus infection found no cases of malnutrition and observed overweight without obesity in 6%, figures similar to those in children without DS (13). The average age of children with DS in the aforementioned study was 26 months, and only 15.5% of the children with congenital heart disease experienced hemodynamic compromise, contrasting with the younger age (8 months) and higher incidence of heart failure (58%) in our study population. Younger age and increased heart failure frequency may explain the higher incidence of underweight status (60.5%). Children with DS are known to have a higher incidence of low weight in the first 2 years of life and excess weight in older age (41). These clinical features, along with the child's critical condition, are likely to be similar to those seen in other PICUs. Nevertheless, given the limited number of children with DS, our results should be interpreted with caution.

The lack of consensus on appropriate reference standards for assessing the nutritional status of children with DS poses a challenge when comparing published studies. Exclusive use of specific growth charts has been questioned (41), advocating for CDC charts in older children (23). One study found overweight or obesity rates of 43%, 57%, and 66% using specific, NCHS, and WHO charts, respectively (42). Applying a DS specific growth chart for cardiac patients under 18 years old revealed a higher frequency of underweight than overweight status (43), possibly due to their cardiac condition. We applied specific charts for children under 1 year and WHO charts for older children (22). Regardless of the criteria used, uniform evaluation standards are essential for proper study comparisons.

Most PO patient outcomes were favorable, possibly due to our definition of what is considered PO status. All patients were relatively stable pre-surgery, with most requiring MV only as transient support. Conversely, children with respiratory issues exhibited longer DMV and fewer VFDs, likely due to their underlying diagnoses; longer hospital stays appear linked to longer DMV, as previously noted.

Our study has several limitations. It was conducted at a single center, with analysis of 38 DS children representing a relatively small cohort. Anthropometry for critically ill patient nutritional assessment is questionable due to physiological instability, fluid management alterations, and presence of edema (30), although we attempted to mitigate this effect by excluding clinically edematous children. DS is a lifelong condition, and our follow-up was limited to hospital course, thus post-discharge outcomes are unknown. Center-specific factors such as PICU bed availability may have influenced hospitalization duration.

Our findings may not generalize to other PICUs with different types of patients, diagnoses, and support availability. PICUs including children undergoing cardiac surgery may experience different outcomes compared to ours. Nonetheless, we believe our study reveals trends in a highly vulnerable population increasingly present in critical care settings globally. Given the heightened susceptibility to infections and increased risk of severe infections in children with DS (16, 18), maintaining up-to-date immunizations against common pathogens, including those for emerging viruses such as COVID-19 (5, 37), is recommended.

In conclusion, children with DS admitted to our PICU exhibited higher frequencies of being underweight, increased mortality risk, greater incidence of HAIs, longer hospital stay, and fewer VFDs compared to children without DS. HAIs and DMV were independently associated with DS. Although some recent findings open new immunological therapy strategies (16), to date there are no specific therapies that have been useful in these children, which is why they require close monitoring to promptly detect complications during their course, particularly if the acute illness is infectious in origin.

## Data Availability

The raw data supporting the conclusions of this article will be made available by the authors, without undue reservation.
